# Turning the Old Adjuvant from Gel to Nanoparticles to Amplify CD8^+^ T Cell Responses

**DOI:** 10.1002/advs.201700426

**Published:** 2017-11-09

**Authors:** Hao Jiang, Qin Wang, Lin Li, Qin Zeng, Hanmei Li, Tao Gong, Zhirong Zhang, Xun Sun

**Affiliations:** ^1^ Key Laboratory of Drug Targeting and Drug Delivery Systems Ministry of Education West China School of Pharmacy Sichuan University No.17, Block 3, Southern Renmin Road Chengdu 610041 P. R. China

**Keywords:** aluminum hydroxide, antigen presenting cell, immune response, lymph nodes, nanoparticles

## Abstract

Due to its safety and efficacy, aluminum hydroxide is used as an immune adjuvant in human vaccines for over 80 years. Being a Th2 stimulator, the classical gel‐like adjuvant, however, fails to generate CD8^+^ T cell responses, which are important for cancer vaccines. Here, aluminum hydroxide is turned from gel into nano‐sized vaccine carriers AlO(OH)‐polymer nanoparticles (APNs) to promote their lymphatic migration. After actively uptaken via scavenger receptor‐A by antigen‐presenting cells (APCs) resident in lymph nodes (LNs), APNs destabilize lysosomes resulting in efficient cytosolic delivery and cross‐presentation of antigens. It is demonstrated that administration of APNs loaded with ovalbumin (OVA) and CpG led to the codelivery of both cargos into APCs in LNs, leading to their activation and subsequent adaptive immunity. A prime‐boost strategy with low doses of OVA (1.5 µg) and CpG (0.45 µg) induces potent CD8^+^ T cell responses and dramatically prolongs the survival of B16‐OVA tumor‐bearing mice. More impressively, when using B16F10 lysates instead of OVA as antigen, substantial antitumor effects on B16F10 tumor model are observed by using APN‐CpG. These results suggest the great potential of APNs as vaccine carriers that activate CD8^+^ T cell responses and the bright prospect of aluminum adjuvant in a nanoparticle formulation.

## 1 Introduction

Vaccines are the most cost‐effective life‐saving technologies for protecting people from infectious diseases.^1^ However, the development of vaccines against major global diseases such as cancers, AIDS, tuberculosis, and malaria remains a great challenge.^2^ Cellular immune responses, especially strong CD8^+^ T cell responses, are essential to treat these diseases.^1^ Using polymers to package antigen and adjuvant into nanosized vehicles is a prospective strategy to improve immune responses.^3^, ^4^, ^5^, ^6^ Various nanosized vaccine carriers have been developed in recent years, but their clinical translation remains very slow due to complex regulatory requirements. Here, we aim to maximize the adjuvantity of the time‐honored adjuvant aluminum hydroxide, by turning it from gel into nano‐sized vaccine carriers. Aluminum hydroxide as the most widely used adjuvant for humans has shown good safety and efficacy in numerous licensed vaccines over 80 years.^7^ However, the name of aluminum hydroxide does not describe the structure of the adjuvant. X‐ray diffraction and infrared spectroscopy identified aluminum hydroxide adjuvant more exactly as crystalline aluminum oxyhydroxide, [AlO(OH)].^8^ As a vaccine carrier, the positively charged AlO(OH) can easily adsorb negatively charged antigens or other adjuvants via electrostatic interactions,^9^ as well as “ligand exchange” of phosphate groups from antigens or adjuvants with surface hydroxyl groups from AlO(OH).^10^ The time‐tested safety profile and easy interaction with antigen and other adjuvants make AlO(OH) more advantageous in vaccine delivery than other inorganic materials, such as calcium phosphate^11^ and porous silicon.^12^ For vaccines that aim to induce CD8^+^ T cell response, a major challenge is that CD8^+^ T cell training needs MHC‐I presentation, but exogenous antigens are usually processed for MHC‐II presentation.^13^ This can be improved by dendritic cell (DC)‐mediated cross‐presentation. Two main intracellular pathways, “cytosolic” and “vacuolar,” have been reported for the cross‐presentation of exogenous antigens by DCs.^13^ For the “cytosolic” pathway, antigen degradation takes place in the cytosol. However, nanoparticles are normally phagocytosed into endosomes and the escape of antigen from endosomes or lysosomes into the cytosol is not efficient.^14^ An earlier report showed that AlO(OH) taken up by APCs could induce lysosomal swelling and damage,^15^ which indicated AlO(OH)‐based nanoparticles might be beneficial to lysosomal escape, cytosolic delivery and cross‐presentation. Nevertheless, turning AlO(OH) into nanoparticles for lymph node targeted delivery has never been reported.

Mixing AlO(OH) with antigen may reduce the rate of antigen elimination from the injection site (usually as peripheral vaccination) and prolong contact between the antigen and immune system, resulting in stronger immune responses than antigen alone.^16^ However, immature peripheral APCs, especially DCs, are present in extremely low numbers in peripheral tissues. This can limit APC activation and T cell responses.^17^ Studies have proven that lymph nodes contain a large number of phagocytically active DCs, which has aroused ever‐increasing interests in targeting lymph nodes to enhance immune responses.^6^, ^17^ The size of vehicles is the most important factor for lymph node traffic. After subcutaneous or intradermal injection, vehicles sized between 10 and 100 nm preferentially enter lymphatic capillaries rather than blood capillaries due to structural differences between blood and lymphatic capillaries in interstitium.^18^, ^19^ Although AlO(OH) is commonly aggregated in the range from 1 to 20 µm in diameter, it actually consists of small primary fibers with an average size of 4.5 × 2.2 × 10 nm,^8^ which may be amenable to nanoscale control for efficient lymph node delivery. Nevertheless, transporting antigens and adjuvants to lymph nodes do not necessarily guarantee the subsequent immune responses. Antigen and adjuvant may simply pass around the outside of the lymph node via the subcapsular sinus and leave directly via the efferent lymph,^18^ resulting in low uptake by APCs and weak activation of T and B cells. Thus, lymph node retention is also important for generating immune responses. A more robust strategy for triggering adaptive immunity is to target the nanovehicle directly to APCs within lymph nodes. This poses a challenge, because the properties that enhance cellular uptake of vehicles usually are unfavorable to lymph node drainage. For example, bearing positive charges or being larger than 100 nm promotes particle internalization by APCs, but makes them difficult to enter lymphatic capillaries.^18^ Thus, it is difficult to achieve both efficient lymphatic drainage and subsequent internalization by APCs. Ligand modification of the vehicles should be able to facilitate both processes in principle, but results so far have been less than impressive.^20^ In addition, ligand modification increases the production cost and complexity of vehicles, making their in vivo behavior more difficult to predict and manage.

Here, this work aims to explore using AlO(OH)‐based nanoparticles to codeliver antigen and adjuvant to lymph node‐resident APCs in order to induce CD8^+^ T cell response. Stabilized by a polyethylene glycol (PEG)‐containing polymer, AlO(OH) nanoparticles maintained an average size below 90 nm to achieve lymph node targeted delivery, and showed efficient internalization by APCs without any ligand or antibody modification. Loaded with antigen and adjuvant, these AlO(OH) nanoparticles induced potent CD8^+^ T cell response in vivo, and significantly inhibited the growth of malignant melanoma.

## 2 Results and Discussion

### 2.1 APNs Are Efficiently Internalized by APCs

**Figure 1:**
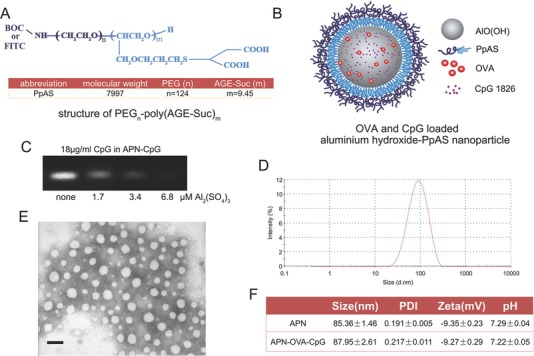
APN structure and morphology. A) Chemical structure of the PEG‐poly(AGE‐Suc) polymer. B) Schematic representation of APNs, in which OVA and CpG are adsorbed by aluminum hydroxide. C) Agarose gel electrophoresis showed CpG completely encapsulated by APNs at 6.8 × 10^−6^ m Al_2_(SO_4_)_3_. D) Representative size distribution of APNs based on dynamic light scattering. E) Representative transmission electron image of APNs, showing approximately spherical nanoparticles with a diameter <80 nm. Bar, 100 nm. F) Size, polydispersity index, zeta potential, and pH of APN and APN‐OVA‐CpG. Data are shown as mean ± SEM (*n* = 4). AGE, allyl glycidyl ether; Suc, mercaptosuccinic acids; BOC, tert‐butyloxycarbonyl; FITC, fluorescein isothiocyanate; CpG‐ODN, CpG oligonucleotide; OVA, ovalbumin.

AlO(OH) on its own cannot be delivered efficiently to lymph nodes because of its large size and positive charge at neutral pH (Figure S1A, Supporting Information). These properties facilitate interactions with proteins and cell membranes, causing the adjuvant to remain at the site of injection.^21^, ^22^, ^23^ To shield the positive charges of AlO(OH), we prepared AlO(OH)‐polymer nanoparticles (APNs) easily by a vortex using a wheat‐like PEG derivative PEG‐poly(AGE‐Suc) (PpAS) previously synthesized in our group (**Figure** **1**A,B).^24^ We successfully loaded ovalbumin (OVA) and CpG into APNs and found that increasing Al_2_(SO4)_3_ concentration to 6.8 × 10^−6^ m led to the complete entrapment of CpG (Figure 1C; Figure S1B, Supporting Information). Incorporation of OVA and CpG did not significantly affect the size or zeta potential of APNs (Figure 1F). The zeta potential of APNs was −9.35 ± 0.23 mV at pH 7.29 (Figure 1F), indicating that the polymer shielded the positive charges of AlO(OH) (Figure S1A, Supporting Information). APNs are negative charged and had a diameter less than 90 nm (Figure 1D–F). More importantly, not like those agglomerative mineral nanoparticles, APNs are monodispersed in aqueous solution (Figure 1E). All these properties afford APNs great potential for lymph node trafficking.^18^

**Figure 2:**
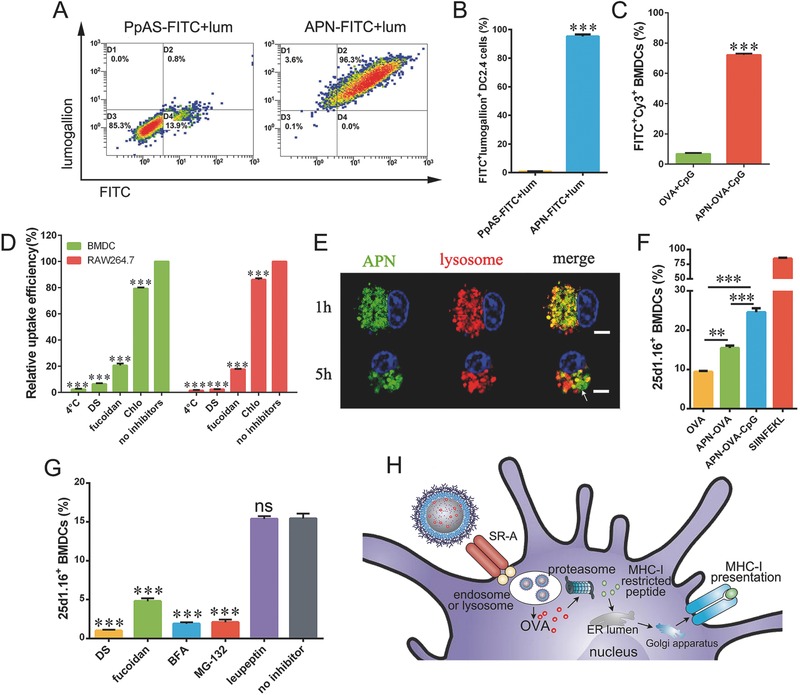
APNs promote OVA internalization and cross‐presentation in APCs. DC2.4 cells were cultured for 1 h at 37 °C with APNs doubly labeled with FITC and lumogallion. Dot plots were gated on all visible cells; values indicate the percentages of cells detected in each gate. A) Representative data from one sample per group are shown. B) Quantitation of percentages of DC2.4 cells positive for both FITC and lumogallion based on the plots in panel (A). C) BMDCs were cultured for 1 h with APNs loaded with FITC‐OVA and Cy3‐CpG. The results show codelivery of OVA and CpG to BMDCs. D) Cultures of DC2.4 or RAW264.7 cells were preincubated for 1 h in medium without inhibitor (no inhibitor) or containing dextran sulfate (DS), fucoidan, or chloropromazin (Chlo). Next FITC‐APN was added to the cultures, which were incubated for 1 h, then harvested and analyzed by flow cytometry. In energy‐depletion experiments, cultures were incubated at 4 °C from the preincubation step until harvest. E) Representative confocal laser scanning images showing BMDCs cultured for different periods with FITC‐APN (green); lysosomes were stained with LysoTracker Red (red). Nonoverlapping green and red signals showed lysosomal escape of APN (white arrow). Bar, 5 µm. F) APN‐OVA and APN‐OVA‐CpG significantly promoted cross‐presentation of OVA in BMDCs at 37 °C. G) BMDCs were pretreated with the indicated inhibitors for 1 h, then APN‐OVA was added and cells were incubated a further 12 h. Cells were harvested and analyzed by flow cytometry. Pretreatment with DS or fucoidan reduced the levels of H2‐K^b^‐SIINFEKL complexes; both these compounds block APN‐OVA uptake. Similar results were observed after pretreatment with brefeldin A (BFA), which blocks ER–Golgi membrane trafficking, or MG‐132, which inhibits the proteasome. Leupeptin, which inhibits cysteine proteases, did not affect surface levels of H2‐K^b^‐SIINFEKL complexes. H) Schematic of APNs promotes antigen cross‐presentation via proteasome‐dependent “cytosolic” pathway in BMDCs. Data are shown as mean ± SEM of 4–5 replicates per experiment in three independent experiments. Each treatment condition was compared with the “no inhibitor” condition using one‐way ANOVA, D,G) followed by the Dunnett post‐test or B,C) using the two‐tailed Student's *t* test. *P < 0.05; **P < 0.01; ***P < 0.001; ns, not significant.

Next we exposed cultures of DC2.4 dendritic cells, bone marrow‐derived dendritic cells (BMDCs) or RAW264.7 macrophages to APNs and observed high internalization, based on fluorescein isothiocyanate (FITC) labeling or lumogallion staining of APNs (Figure S2, Supporting Information). Lumogallion can track aluminum adjuvants that have been phagocytosed by cells.^25^ When cultures were exposed to APNs labeled using both PpAS‐FTIC and lumogallion, the cells showed strong, double signal for FITC and lumogallion by flow cytometry. This result suggested that APNs were phagocytosed as a whole rather than separately as AlO(OH) and PpAS (**Figure** **2**A,B), given that PpAS alone was not taken up by DCs (Figure S2A, Supporting Information). Then, we loaded APNs with FITC‐labeled OVA and Cy3‐labeled CpG and examined their internalization by BMDCs. Internalization of APN‐OVA‐CpG was significantly higher than that of free OVA and CpG added to cells (Figure 2C), suggesting that encapsulating OVA and CpG into APNs promoted their couptake by DCs.

To explore the underlying mechanism of APN internalization, uptake experiments were performed in the presence of different inhibitors that block a certain endocytotic pathway. APN uptake by DC2.4 and RAW264.7 cells became markedly less efficient when the cultures were incubated at 4 °C or pretreated with dextran sulfate, fucoidan or chlorpromazine (*P* < 0.001, Figure 2D). Dextran sulfate and fucoidan, both of which are ligands of scavenger receptor A,^26^, ^27^ almost completely abrogated endocytosis. This suggests that APN internalization involves scavenger receptor A‐mediated endocytosis. The inhibition of endocytosis caused by 4 °C and chlorpromazine respectively suggests that an energy‐dependent process and clathrin‐mediated endocytosis also contribute to APN internalization.^28^ The other inhibitors did not significantly inhibit endocytosis (Figure S3A, Supporting Information). Interestingly, this scavenger receptor A‐mediated internalization depended on the AlO(OH) core of APN, because replacing AlO(OH) with calcium phosphate (CaP) drastically reduced the internalization efficiency (Figure S3B, Supporting Information), even though the size and zeta potential of CaP‐polymer nanoparticles were similar to those of APNs (Figure S3C, Supporting Information). An earlier research reported the high affinity of alum for membrane lipids of DCs.^29^ We speculate that the high internalization rate of APN on DCs may be related to this property.

### 2.2 APNs Promote Cross‐Presentation of OVA in BMDCs

We then investigated the abilities of APNs on cytosolic delivery and DC cross‐presentation. When using LysoTracker Red to visualize lysosomes of BMDCs, separated fluorescence of APNs (green) and lysosomes (red) were observed, which suggested successful lysosomal escape and cytosolic delivery of APNs (Figure 2E). This was consistent with the earlier report that AlO(OH) can destabilize lysosomes.^15^ H2‐K^b^‐SIINFEKL complexes on the surface of BMDCs were detected using the monoclonal antibody 25d1.16 and flow cytometry. Taking free synthetic SIINFEKL peptide as a positive control, BMDCs were exposed to SIINFEKL peptide, OVA, APN‐OVA or APN‐OVA‐CpG and incubated at 4 or 37 °C. The H2‐Kb‐SIINFEKL complexes on cell surface were detected by flow cytometry. The proportion of H2‐K^b^‐SIINFEKL positive cells was 40.5% for cells treated with free SIINFEKL peptide at 4 °C and the ratios were much lower (≈5%) for cells treated with OVA, APN‐OVA or APN‐OVA‐CpG (Figure S4, Supporting Information). This result suggested that no obvious cross‐presentation of OVA was observed when internalization was inhibited by the low temperature. Nervertheless, APN‐OVA significantly increased the proportion of 25d1.16 positive cells by about 1.7‐fold compared with free OVA at 37 °C (Figure 2F). These results suggest that APNs improved OVA processing and promoted cross‐presentation. Loading APNs with CpG as well as OVA further increased staining. The proportion of positive cells treated with APN‐OVA‐CpG was higher than those treated with APN‐OVA (*P* < 0.001, Figure 2F). This is likely due to the fact that antigen presentation mainly occurs in mature DCs and CpG can promote the maturation of BMDCs.

Then, we applied inhibitors of antigen uptake and intracellular trafficking to elucidate the cross‐presentation pathway after APN‐OVA internalization. To confirm that cross‐presentation was dependant on APN‐OVA uptake, we pretreated BMDCs with dextran sulfate and fucoidan, which strongly inhibited cellular uptake (Figure 2G). As expected, both dextran sulfate and fucoidan reduced the proportion of cells staining positive for H2‐K^b^‐SIINFEKL complexes (Figure 2G). These results confirm that active uptake mechanisms contribute to cross‐presentation. To investigate which pathway (“cytosolic” or “vacuolar”) may participate in APN‐OVA cross‐presentation, we treated BMDCs with brefeldin A (BFA), which inhibits protein transport from the endoplasmic reticulum to Golgi;^30^ MG‐132, which inhibits the proteasome;^31^ or leupeptin, which inhibits cysteine proteases.^32^ Pretreating BMDCs with BFA or MG‐132, but not leupeptin, led to significantly lower proportions of cells staining positive for H2‐K^b^‐SIINFEKL complexes than the proportion observed in the absence of any inhibitor (*P* < 0.001, Figure 2G). These results together with Figure 2E suggest that the cytosolic pathway, but not the vacuolar one, drives APN‐OVA cross‐presentation by BMDCs (Figure 2H).

### 2.3 APN‐CpG Efficiently Activates APCs in Draining Lymph Nodes

**Figure 3:**
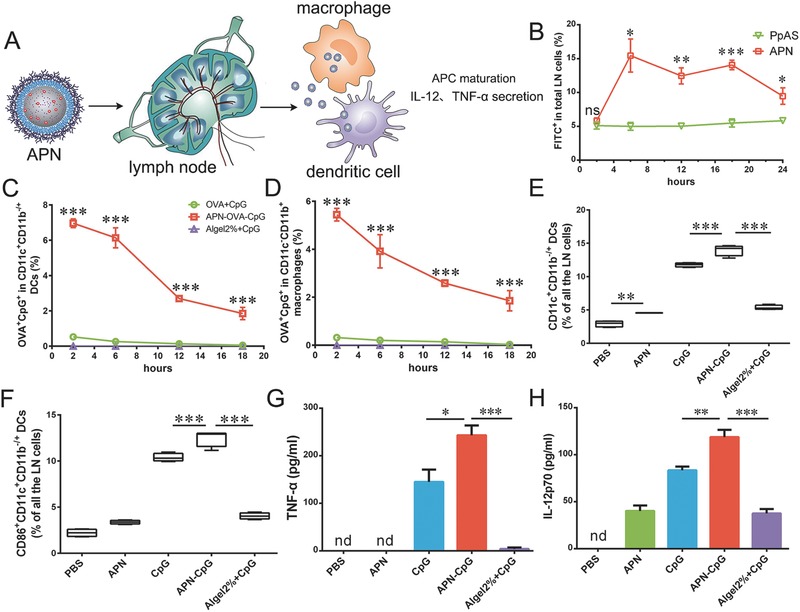
APN‐CpG activates APCs in the draining lymph nodes in vivo. A) Schematic of APN‐CpG activates DCs and marcophages in lymph nodes. C57BL/6 mice were injected in the footpad with 25 µL of the indicated formulations, and popliteal lymph nodes were isolated for analysis at the indicated time points. Total lymph node cell populations were gated and analyzed using flow cytometry to determine the percentage of cells positive for B) FITC‐APN. After cells were stained according to subtype, flow cytometry was used to determine the percentages of OVA‐ and CpG‐positive cells in C) CD11c^+^CD11b^−/+^ DCs or D) CD11c^−^CD11b^+^ macrophages. C57BL/6 mice were immunized by footpad injection with APNs (with or without 0.45 µg CpG), 0.45 µg free CpG or Algel2% with 0.45 µg CpG. At 3 d after injection, draining lymph nodes were isolated and the percentages of total E) CD11c^+^CD11b^−/+^ DCs and F) mature CD11c^+^CD11b^−/+^ DCs, based on surface expression of CD86, in total lymph node cells were determined using flow cytometry. Lymph nodes draining the site of administration were harvested 3 d after injection and homogenized into a cell suspension that was cultured for 8 h and then evaluated for expression of G) TNF‐α and H) IL‐12p70 using ELISA. Box plots in panels (E) and (F) indicate medians and 95% confidence intervals. Data in other panels are shown as mean ± SEM. The indicated pairwise comparisons in panels (B)–(D) were assessed for significance at each time point using the two‐tailed Student's *t* test. *P < 0.05; **P < 0.01; ***P < 0.001; ns, not significant; nd, not detectable. Two independent experiments were performed using four mice per group.

Next, we explored the potential of APNs to activate APCs in lymph nodes (**Figure** **3**A). Vehicle size is critical here, since particles with a size of 10–100 nm enter the lymph system more efficiently than larger particles, but they are retained to a smaller extent in lymph nodes.^18^, ^33^ Impressively, ≈85 nm APNs not only reached draining lymph nodes efficiently but also remained there at a relatively high concentration for more than 24 h (Figure 3B). Since maximizing the immune response requires delivering antigen and adjuvant to the same APC,^34^ we explored whether OVA and CpG were cointernalized in lymph node‐resident DCs (CD11c^+^CD11b^+/−^) or macrophages (CD11c^−^CD11b^+^) after delivered by APNs. Injecting animals with APN‐OVA‐CpG led to significantly greater colocalization of OVA and CpG by about 34.7‐fold on DCs (Figure 3C) and 55.7‐fold on macrophages (Figure 3D), compared with OVA + CpG at 18 h. These results suggest that APNs can efficiently deliver cargos to APCs in the lymph nodes. In contrast, after loading OVA and CpG with Algel2%, minimal cointernalized OVA and CpG were observed. This result indicates that OVA and CpG were trapped in the Algel2% “depot” and largely unable to traffic to lymph nodes.^21^

Delivering antigen and adjuvant to APCs in lymph nodes is the first step to elicit T cell immune responses. APCs can prime T cells only after being stimulated by adjuvant and subsequently expressing both costimulatory molecules and inflammatory cytokines. To assess the ability of APNs to activate APCs in lymph nodes, we first compared the ability of APNs or standard alum adjuvant Algel2% to expand the populations of DCs and macrophages. APN‐CpG increased the magnitude of DCs (Figure 3E) and macrophages (Figure S5A, Supporting Information) to a significantly greater extent than free CpG or Algel2% + CpG. In addition, surface markers of DC and macrophage maturation (CD40, CD80, and CD86) were upregulated in animals treated with free CpG or APN‐CpG (Figure 3F; Figure S5B–F, Supporting Information). APN‐CpG also led to significantly higher production of cytokines TNF‐α and IL‐12p70 by 1.7‐fold and 1.4‐fold, respectively, when compared with free CpG, and 63.4‐fold and 3.2‐fold, respectively, when compared with Algel2% + CpG (Figure 3G,H). In contrast to APN‐CpG, Algel2% + CpG led to only moderate activation of macrophages and no appreciable activation of DCs, based on the detected proportions of APCs, the expression of costimulatory molecules and the production of cytokines (Figure 3E–H; Figure S5, Supporting Information). These results may be caused by the inefficient lymph node delivery of Algel2%. Whatever the explanation, the results suggest that APN is a suitable vehicle for delivering CpG to lymph nodes and activating APCs there.

### 2.4 APN‐CpG Induces Potent Antibody and CD8^+^ T Cell Responses

**Figure 4:**
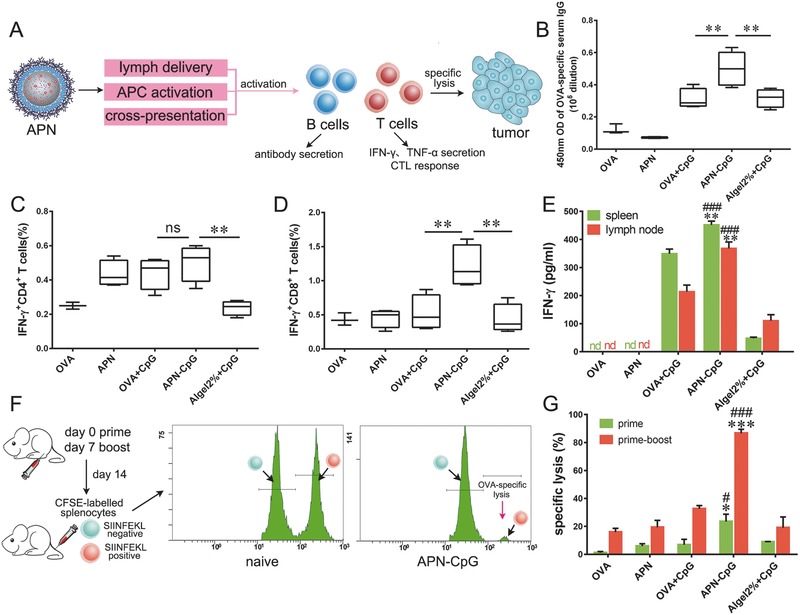
APN‐CpG induces potent cellular immune responses. A) Schematic of APN‐CpG induces antibody and T cell responses. C57BL/6 mice were vaccinated in the footpad on day 0 with APNs (with or without 0.45 µg CpG), 1.5 µg OVA (with or without 0.45 µg CpG), or Algel2% with 0.45 µg CpG. A boost injection was performed on day 7 with the same doses of OVA and CpG. B) Mice were sacrificed on day 14, serum was collected, diluted 10^5^ times, and analyzed for total anti‐OVA IgG. Popliteal lymph nodes and spleens of vaccinated mice were harvested and homogenized into cell suspensions. C,D) To restimulate CD4^+^ and CD8^+^ T cells, splenocytes were incubated for 6 h at 37 °C with, respectively, 100 µg mL^−1^ OVA or 2 µg mL^−1^ SIINFEKL. Brefeldin A (5 µg mL^−1^) was added for the last 5 h of culture. Percentages of IFN‐γ‐expressing C) CD4^+^ and D) CD8^+^ T cells were determined using flow cytometry. E) Lymph node cells and splenocytes were cultured for 60 h in the presence of 2 µg mL^−1^ SIINFEKL, and production of IFN‐γ was determined using ELISA. Panels (D) and (E) show that APN‐CpG activated CD8^+^ T cells more strongly than OVA + CpG or Algel2% + CpG. F) Schematic of the carboxy fluorescein succinimidyl ester (CFSE) labeling method to evaluate the OVA‐specific CTL response induced by APN‐CpG. C57BL/6 mice were vaccinated in the footpad on day 0 and boosted on day 7. On day 7 (for prime only) or 14 (for prime and boost), vaccinated and naive mice were injected with a 1:1 mixture of splenocytes, half of which had been incubated with SIINFEKL peptide and stained with a high level of CFSE, and the other half of which had been stained with a tenfold lower level of CFSE. Spleens were harvested after 18 h, and splenocytes were analyzed by flow cytometry. G) APN‐CpG triggered a potent CTL response. Box plots in panels (B)–(D) show medians and 95% confidence intervals, while the data in other panels are mean ± SEM. In panels (E) and (G), asterisks indicate *P* values associated with comparisons of APN‐CpG and OVA + CpG; pound signs indicate the same *P* ranges for comparisons between APN‐CpG and Algel2% + CpG. *P < 0.05; **P < 0.01; ***P < 0.001; ^#^ *P* < 0.05; ^##^ *P* < 0.01; ns, not significant; nd, not detectable. Experiments were performed three times with 5–6 mice per group.

Based on the above characteristics, we investigated whether APNs could induce potent antigen‐specific immune ressponses (**Figure** **4**A). C57BL/6 mice were vaccinated with APNs (with or without 0.45 µg CpG), 1.5 µg OVA (with or without 0.45 µg CpG), or Algel2% with 0.45 µg CpG. Animals were primed on day 0 and boosted on day 7. First, we test the antibody response generated by different groups. In assays of total IgG, APN‐CpG showed the same titer with Algel2% + CpG (Figure S6A, Supporting Information). But when compared the absorbance in the highest titer, APN‐CpG induced significantly higher absorbance at 450 nm than Algel2% + CpG (*P* < 0.01, Figure 4B). Analysis of IgG antibody subtypes showed that APN‐CpG induced potent both IgG1 and IgG2a responses, indicating a balanced T help (Th) cell response (Figure S6B–D, Supporting Information). In contrast, Algel2% + CpG induced strong IgG1 but weak IgG2a responses, resulting in a Th2‐skewed immunity (Figure S6B–D, Supporting Information).

We next assessed the CD8^+^ T cell response induced by APN‐CpG. Popliteal lymph nodes and spleens of vaccinated mice were harvested on day 14 and homogenized into a cell suspension for ex vivo culture or restimulation. Splenocytes were restimulated ex vivo, and proportions of antigen‐specific, IFN‐γ‐producing CD4^+^ and CD8^+^ T cells were determined. Although splenocytes from mice immunized with APN‐CpG did not show more Th1 cells (IFN‐γ^+^CD4^+^) than those immunized with free OVA + CpG (Figure 4C), APN‐CpG induced a stronger cytotoxic CD8^+^ T cell response than OVA + CpG or Algel2% + CpG, leading to a 2.3‐fold or 2.8‐fold, respectively, higher proportion of IFN‐γ^+^CD8^+^ T cells in spleens (*P* < 0.01, Figure 4D). The production of IFN‐γ (Figure 4E) and TNF‐α (Figure S7, Supporting Information) in lymph nodes and spleens also indicated stronger CD8^+^ T cell responses induced by APN‐CpG than OVA + CpG or Algel2% + CpG.

These experiments suggest that APN‐CpG can induce an effector phenotype in CD8^+^ T cells, but they do not address whether the induction is sufficient to ensure that immune cells efficiently recognize and kill antigen‐bearing target cells. To address this question, we examined whether our vaccine formulations induce cytotoxic T lymphocyte (CTL) activity (Figure 4F). SIINFEKL‐stimulated CFSE^high^‐labeled splenocytes were mixed 1:1 with untreated CFSE^low^‐labeled splenocytes and injected into mice vaccinated with various formulations.^35^ In experiments involving only prime immunization or both prime and boost immunization, APN‐CpG led to a significantly higher percentage of target cell lysis than OVA + CpG or Algel2% + CpG (Figure 4G). When examined the OVA‐specific lysis on day 14 (prime and boost), APN‐CpG killed much more target cells by 2.7‐fold than OVA + CpG and 4.5‐fold than Algel2% + CpG (Figure 4G).

### 2.5 APN‐CpG Inhibits the Growth of B16‐OVA and B16F10 Tumors

**Figure 5:**
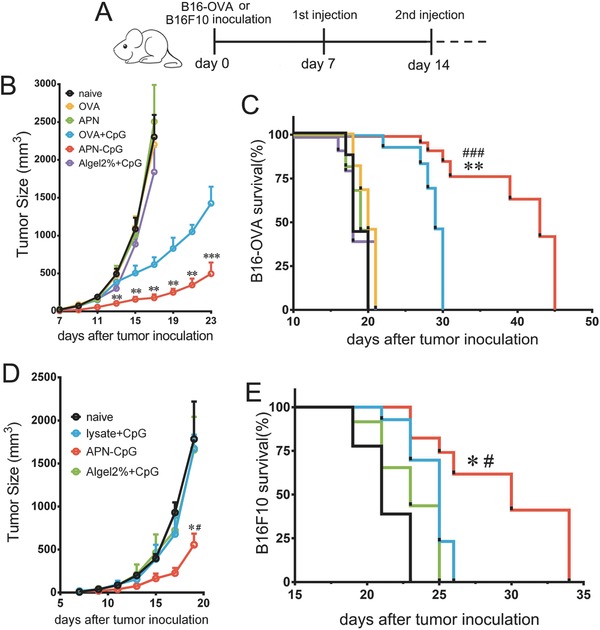
APN‐CpG shows antitumor activity in therapeutic melanoma models. A) B16‐OVA or B16F10 cells (5 × 10^5^) were subcutaneously implanted into the flank of mice on day 0, and the tumors were allowed to grow for 7 d to a size of about 30–40 mm^3^. The first therapeutic injection was administered on the footpad on day 7 with APNs (with or without 0.45 µg CpG), 1.5 µg OVA or B16F10 lysates (with or without 0.45 µg CpG), or Algel2% with 0.45 µg CpG. The second injection was performed on day 14 with the same doses of antigen and CpG. APN‐CpG was associated with B) significantly slower tumor growth than OVA + CpG and Agel2% + CpG, C) as well as significantly longer survival. In the more aggressive B16F10 model, mice treated with APN‐CpG also showed D) significantly slower tumor growth than lysate + CpG and Agel2% + CpG (D), E) as well as significantly longer survival. Tumor growth curves (mean ± SEM) are shown together with Kaplan–Meier survival curves. Results in panels (B) and (D) for groups treated with APN‐CpG or OVA + CpG (lysate + CpG in D) were compared at each time point using the two‐tailed Student's *t*‐test. Survival results in panels (C) and (E) were compared between indicated groups using a log‐rank test, asterisk indicates *P* values associated with comparison of APN‐CpG and OVA + CpG (lysate + CpG in panel (D); pound sign indicates the same *P* ranges for comparison between APN‐CpG and Algel2% + CpG. *P < 0.05; ^#^ *P* < 0.05; ^##^ *P* < 0.01; ^###^ *P* < 0.001; ns, not significant.

IFN‐γ is a critical effector molecule to tumour rejection. IFN‐γ can impede tumour growth by acting directly on cancer cells;^36^ or induced regression of the tumour vasculature, resulting in arrest of blood flow and subsequent collapse of tumours;^37^ or drives the fragility of surrounding Tregs, boosts antitumor immunity, and facilitates tumor clearance.^38^ The high production of IFN‐γ induced by APN‐CpG (Figure 4E) enlightened us that APN‐CpG could be a valuable vector for antitumor vaccines. Next, we investigated whether antigen‐specific responses induced by APN‐CpG are potent enough to eradicate established tumors. C57BL/6 mice were implanted with 5 × 10^5^ B16‐OVA cells, and tumors were allowed to grow for 7 d to a size of about 30–40 mm. Then mice were given a priming injection with 1.5 µg OVA and 0.45 µg CpG on day 7, followed by a boost with the same doses on day 14 (**Figure** **5**A). Only mice immunized with APN‐CpG or OVA + CpG delayed tumor onset and prolonged survival (Figure 5B,C); mice treated with other formulations, including Algel2% + CpG, showed no obvious differences from the naïve control group. At all time points after day 15, APN‐CpG was associated with significantly slower tumor growth than OVA + CpG (Figure 5B) as well as significantly longer survival (Figure 5C). Further, we chose B16F10 lysates instead of OVA as antigen and studied the therapeutic effect of APN‐CpG in murine B16F10 melanoma. We used the same therapeutic schedule with B16‐OVA tumor. C57BL/6 mice were implanted with 5 × 10^5^ B16F10 cells on day 0. Then, 1.5 µg B16F10 lysates and 0.45 µg CpG were injected on day 7 and day 14. Inspiringly, even we utilized quite low doses antigen (1.5 µg lysates) and adjuvant (0.45 µg CpG), APN‐CpG significantly inhibited tumor growth and prolonged survival than lysate + CpG and Algel2% + CpG (Figure 5D,E). Unlike in B16‐OVA tumor model, free antigen combined with CpG (lysate + CpG) failed to inhibit tumor growth and improve survival. As a matter of fact, it showed no differences in tumor growth from the naïve control and similar survival with Algel2% + CpG (Figure 5D,E). These results provide direct evidence that APN‐CpG can treat tumors by boosting antitumor immunity.

Delivering both antigen and adjuvant to the same APCs is critical to induce maximal antigen‐specific immune responses.^34^, ^39^, ^40^ Using suitable vehicles to coencapsulate antigen and adjuvant is a popular and effective strategy. Many materials have been used to package antigens and adjuvants into nanoparticles, micelles, and other vehicles. Studies confirmed the ability of these vehicles to improve immune responses.^3^, ^4^, ^34^, ^41^ In the present work, we focused on the old adjuvant AlO(OH), which widely used in human vaccines for over 80 years because of its safety and efficacy.^7^ AlO(OH) adjuvant is usually used only with bacterial and viral vaccines since they are Th2 stimulators, and such vaccines are intended to generate neutralizing antibodies.^1^, ^16^ Antitumor and some antiviral vaccines, however, aim to induce antigen‐specific CD8^+^ T cell responses, which AlO(OH) adjuvant do not appear to affect. Here, we applied AlO(OH) adjuvant in a novel way: as a lymph node delivery nanoparticle formulation. Using a PEG‐derivative PpAS polymer, we successfully controlled the aggregation of AlO(OH) and formulated APNs with a diameter of 85.36 ± 1.46 nm (Figure 1F). This small size and PEG coating allowed the APNs to traffick efficiently to the draining lymph nodes and to retain in the lymph nodes (Figure 3B). Even though a PEG coating and negative charges usually inhibit vehicle uptake, our APNs were efficiently internalized by DCs and macrophages via a process mediated by scavenger receptor‐A (Figure 2D; Figure S2, Supporting Information).

Since AlO(OH) adjuvant can adsorb antigens via electrostatic interactions^9^ and “ligand exchange,”^10^ we were able to load OVA and CpG easily into our APNs. In fact, CpG was completely encapsulated into APNs (Figure 1C) and unencapsulated OVA was removed by ultrafiltration. Coloading OVA and CpG did not affect APN size or zeta potential (Figure 1F), and it ensured the codelivery of antigen and adjuvant to the same APCs, which is crucial for generating the most potent antigen‐specific immune responses. APNs reached draining lymph nodes quickly and remained there at a relatively high concentration for more than 24 h (Figure 3B). In contrast, the percentages of DCs and macrophages that had cointernalized OVA and CpG decreased over time (Figure 3C,D). This decrease despite a stable population of APNs in lymph nodes (Figure 3B) may be because APNs were captured by DCs and macrophages at early time points but OVA was processed by APCs and lost its fluorescence labeling at late time points. Further studies are needed to explore this phenomenon.

In order for exogenous protein antigen to induce CD8^+^ T cell responses, it must be cross‐presented. We found that APNs promoted antigen cross‐presentation by BMDCs, and this strongly depended on temperature and the activity of intracellular antigen transporters as well as the proteasome (Figure 2G). These results suggest that BMDCs cross‐present APN‐trapped OVA via a cytosolic pathway (Figure 2H). Costimulatory molecules expressed on the surface of APCs are important for stimulating naïve T cells and inducing cell‐mediated immunity. CpG‐loaded APNs upregulated the maturation markers CD40, CD80, and CD86 on the surface of DCs and macrophages in lymph nodes (Figure 3F; Figure S5B–F, Supporting Information). In contract to our APNs, the commercial adjuvant Algel2% activated neither DCs nor macrophages under our experimental conditions, even when combined with CpG (Figure 3F; Figure S5B–F, Supporting Information). This may be because CpG associated with Algel2% was unable to reach lymph nodes as most of the Algel2% was retained at the injection site.

Helper CD4^+^ T cells play an important role in initiating cellular immunity by triggering the differentiation of cytotoxic CD8^+^ T cells, and in initiating humoral immunity by stimulating B cells to produce antibodies.^41^ At the antigen and adjuvant doses in our experiments, treatment of mice with APN‐CpG led to a significantly greater proportion of CD8^+^ T cells secreting IFN‐γ than treatment with OVA + CpG or Algel2% + CpG (Figure 4D), even though the treatments APN‐CpG and OVA + CpG led to similar proportions of CD4^+^ T cells secreting IFN‐γ (Figure 4C). APN‐CpG also induced greater IFN‐γ secretion by lymph node cells and splenocytes than the other treatments did (Figure 4E). These results are consistent with IgG antibody subtype studies (Figure S6, Supporting Information) suggesting that APN‐CpG induced much stronger Th1‐type responses than Algel2% + CpG did. A successful vaccine should be able to induce not only effector phenotypes in CD8^+^ T cells but also a CTL response that can recognize and kill antigen‐bearing target cells. APN‐CpG induced a potent CTL response that killed SIINEFKL‐bearing splenocytes (Figure 4G).

The therapeutic efficacy of this CTL response was confirmed in a mouse B16‐OVA model. APN‐CpG containing only 1.5 µg OVA and 0.45 µg CpG (per injection), delivered in two injections, effectively delayed B16‐OVA tumor growth and prolonged survival (Figure 5B,C). We further investigated B16F10 lysates with APNs in an aggressive and poorly immunogenic B16F10 tumor. Inspiringly, APN‐CpG also showed significantly slower tumor growth and longer survival than the mixture formulation (lysate + CpG) and the standard alum adjuvant (Algel2% + CpG) (Figure 5D,E). These efficacies likely reflect the ability of APNs to codeliver the antigen and adjuvant to the same APCs in lymph nodes and promote the cross‐presentation of antigen. Although OVA + CpG exhibited tumor growth inhibition in B16‐OVA tumor model, free tumor lysates and CpG mixture formulation failed to inhibit B16F10 tumor growth. In fact, it showed no inhibition in tumor growth and barely longer survival than the naïve control (Figure 5D,E). This may be because of that the immunogenicity of tumor lysates is much lower than OVA. When compared with OVA antigen, APNs carried tumor lysate antigen and CpG exhibited weaker tumor growth inhibition and survival improvement. This may be related to the very low doses of lysates and CpG used here. We expect better antitumor effects of APNs through increasing the amount of antigen and adjuvant or combining with other therapeutic approaches.

## 3 Conclusions

In this study, we created stable nanoparticles based on the classical vaccine adjuvant AlO(OH). AlO(OH) nanoparticles are neatly formulated and coloaded with antigen and adjuvant via simple vortex mixing. With an average size distribution below 90 nm, AlO(OH) nanoparticles are negatively charged and monodispersed in aqueous solution to ensure lymph nodes targeted delivery. After reaching lymph nodes, AlO(OH) nanoparticles are efficiently internalized by APCs to promote the activation and cross‐presentation of APCs, thus leading to robust antibody‐ and cell‐based immune responses, including a CTL response capable of slowing tumor growth and prolonging survival. These results highlight the potential of AlO(OH) nanoparticles as vaccine delivery vehicles, and the great prospect of AlO(OH) nanoparticles for clinical applications.

## 4 Experimental Section

*APN Preparations*: To prepare APNs, PEG‐poly(AGE‐Suc) and Al_2_(SO_4_)_3_ were dissolved in ultrapure water. PEG‐poly(AGE‐Suc) solution (110 µL, 20 mg mL^−1^) was added to 2‐[4‐(2‐hydroxyethyl)‐1‐piperazinyl]ethanesulfonic acid (HEPES) (340 µL, 100 × 10^−3^ m, pH 8.0) with gentle stirring, and then Al_2_(SO4)_3_ (540 µL, 6.8 × 10^−3^ m) was added dropwise with strong mixing under a vortex shaker for 1 min at room temperature.

To form FITC‐APN, the tert‐butyloxycarbonyl (BOC) group on PEG‐poly(AGE‐Suc) was removed using trifluoroacetic acid and purified on a PD‐10 desalting column. FITC and PEG‐poly(AGE‐Suc) lacking the BOC group were mixed in a molar ratio of 10:1 in carbonate buffer solution (pH 9.6). After stirring overnight at room temperature, unbound FITC was removed by dialysis against ultrapure water for 3 d using a membrane with a 1 kDa molecular weight cutoff. The purified FITC‐PEG‐poly(AGE‐Suc) was lyophilized and used to prepare FITC‐APNs as described above for PEG‐poly(AGE‐Suc).

To form lumogallion‐stained APNs, APNs were prepared as described above and then incubated with 50 × 10^−6^ m lumogallion^25^ for 1 h at room temperature.

For OVA‐containing APN‐CpG, CpG (18 µg) was added to PEG‐poly(AGE‐Suc) HEPES solution and OVA (90 µg) was mixed with Al_2_(SO_4_)_3_ solution; then the HEPES solution and Al_2_(SO_4_)_3_ solution were mixed as described above. OVA‐containing APN‐CpG was further purified by ultrafiltration, and amounts of OVA and CpG were assayed, respectively, using the BCA assay kit (Pierce) and electrophoresis on an agarose gel (1%) stained with GoldView (Generay Biotech).

Algel2% was prepared according to the manufacturer's instructions. Briefly, Alhydrogel 2% (InvivoGen) was added to OVA and CpG solution at a volume ratio of 1:1. The mixture was pipetted up and down for ≈15 min to allow Alhydrogel 2% to effectively adsorb the OVA and CpG.

*Assays of APN Uptake Mechanisms*: To explore the possible mechanisms of APN internalization by APCs, cultures of RAW264.7, DC2.4 or BMDCs were pretreated at 37 °C for 1 h with selective inhibitors of different internalization pathways. Then, APN‐FITC was added and cultures were incubated for a further 1 h. The following inhibitors were used: chlorpromazine (20 × 10^−6^ m), dextran sulfate (100 µg mL^−1^), fucoidan (100 µg mL^−1^), LY294002 (50 × 10^−6^ m), dynasore (50 × 10^−6^ m), amiloride (100 × 10^−6^ m), nystatin (25 × 10^−6^ m), poly‐l‐lysine (200 µg mL^−1^), and methyl‐β‐cyclodextrin (10 × 10^−6^ m). These experiments were repeated at 4 °C to investigate the effect of temperature on APN uptake.

*In Vitro Assays of OVA Cross‐Presentation*: BMDCs were generated from mouse bone marrow as described previously.^42^ On day 8, immature BMDCs were collected and stimulated for 24 h with different formulations, with phosphate‐buffered saline (PBS) as a negative control or with 2 µg lipopolysaccharide (LPS) as a positive control. Expression of costimulatory surface markers of BMDCs was measured by flow cytometry after staining with fluorescent antibodies.

To examine lysosomal escape, BMDCs were incubated for 1, 3, and 5 h with APN‐FITC in confocal dishes. The cells were washed and then incubated with LysoTracker Red (Invitrogen) for 30 min, followed by Hoechst 33342 for 5 min. Cells were then fed fresh medium and observed using a confocal laser scanning microscope.

For cross‐presentation detection, BMDCs were incubated at 37 °C for 2 h with 1.5 µg free OVA, APN‐OVA, APN‐OVA‐CpG (0.45 µg CpG) or 2 µg SIINFEKL peptide. Then the medium was replaced with fresh medium, and cultures were incubated for a further 10 h. Cells were washed twice in PBS, stained using monoclonal antibody 25d1.16 and analyzed using flow cytometry.

To determine the pathway used by BMDCs to cross‐present OVA, we pretreated BMDCs at 37 °C for 1 h with dextran sulfate (100 µg mL^−1^), fucoidan (100 µg mL^−1^), BFA (1 µg mL^−1^), MG‐132 (10 × 10^−6^ m), or leupeptin (100 µg L^−1^). Then APN‐OVA containing 1.5 µg OVA was added and incubated for a further 12 h. BMDCs were collected, washed twice in PBS, stained using the monoclonal antibody 25d1.16, and analyzed using flow cytometry.

*Activation of Bone Marrow‐Derived Dendritic Cells In Vitro*: BMDCs were generated from mouse bone marrow as described previously.^42^ On day 8, immature BMDCs were collected and stimulated for 20 h with different formulations, with PBS as a negative control or with 2 µg LPS as a positive control. Supernatants were collected and analyzed by enzyme‐linked immunosorbent assay (ELISA) for cytokines secretion. Expression of costimulatory surface markers of BMDCs was measured by flow cytometry after staining with fluorescent antibodies.

*Nanoparticle Distribution in Lymph Nodes*: FITC‐OVA and Cy3‐CpG‐contained APNs were injected into the footpad of mice who were sacrificed at 2, 6, 12, and 24 h. Their ipsilateral popliteal draining lymph nodes were harvested, and single‐cell suspensions were prepared by digesting tissues in 1 mg mL^−1^ collagenase D (Roche) for 30 min at 37 °C, and then passing them through a 70 µm cell sieve. Cells were washed with PBS, surface‐stained with anti‐mouse CD11c and CD11b (eBioscience) and analyzed using a flow cytometer.

*Single‐Cell Preparations and Ex Vivo Antigen‐Specific Cell Restimulation*: Immune cells in lymph nodes were obtained by digesting lymph nodes in 1 mg mL^−1^ collagenase D (Roche) for 30 min at 37 °C and disrupting them through a 70 µm cell sieve. Splenocytes were obtained by squeezing the spleen and disrupting the cell aggregates through a cell sieve, and then lysing the red blood cells in ammonium‐chloride‐potassium (ACK) lysing buffer (0.15 m NH_4_Cl, 10.0 × 10^−3^ m KHCO_3_, 0.1 × 10^−3^ m EDTA, pH 7.4). For experiments involving CD8^+^ T‐cell antigen‐specific restimulation and intracellular cytokine staining, immune cells were cultured at 37 °C for 6 h in the presence of 1 µg mL^−1^ SIINFEKL peptide and 2 µg mL^−1^ monensin. For experiments involving CD4^+^ T‐cell antigen‐specific restimulation and intracellular cytokine staining, immune cells were cultured at 37 °C for 6 h in the presence of 100 µg mL^−1^ OVA and 5 µg mL^−1^ brefeldin A. In other experiments, CD8^+^ T cells were cultured in the presence of 1 µg mL^−1^ SIINFEKL, while CD4^+^ T cells were cultured in the presence of 100 µg mL^−1^ OVA for 60 h at 37 °C. In all cases, the culture medium was RPMI‐1640 supplemented with 10% fetal bovine serum (FBS) and 1% penicillin/streptomycin. Then, cells were surface‐ and intracellular‐stained and analyzed by flow cytometry and cytokine levels in the supernatant were assayed using Ready‐SET‐go! ELISA kits (eBioscience).

*Immunization Studies*: All immunizations were performed by footpad injection using a 29‐gauge insulin syringe. Each formulation contained a total of 1.5 µg OVA and 0.45 µg CpG. Mice received priming injections on day 0 and boosters on day 7. Mice were sacrificed on day 14, when blood, draining lymph nodes and spleens were collected for analysis.

Serum levels of anti‐OVA IgG, IgG1, and IgG2a antibodies were determined by ELISA as described.^35^ Serial dilutions from 10^3^ to 10^7^ were performed for each mouse, and absorbance at 450 nm was measured in a microtiter‐plate spectrophotometer.

*Cytotoxic T Lymphocyte Activity In Vivo*: On day 14 after vaccination, mice were injected intravenously with 1 × 10^7^ CFSE‐labeled target cells consisting of 50% SIINFEKL‐pulsed splenocytes (termed “CFSE^high^”) labeled with 4 × 10^−6^ m 5‐(and 6)‐carboxyfluorescein diacetate, succinimidyl ester (CFSE, Invitrogen) and 50% unpulsed splenocytes (“CFSE^low^”) labeled with 0.4 × 10^−6^ m CFSE. Mice were sacrificed 18 h after adoptive transfer, and splenocytes were harvested for analysis by flow cytometry. The percentage of specific lysis was calculated from the formula.^35^

Percentage specific lysis = 100% × [1‐(ratio of CFSE^low^/CFSE^high^ cells recovered from naive mice)/(ratio of CFSE^low^/CFSE^high^ cells recovered from immunized mice)]

*Preparation of Tumor Lysate*: B16F10 cells were lysed by four freeze (liquid nitrogen) and thaw (37 °C water bath) cycles. Larger particles and cell debris were removed by centrifugation (500 g for 10 min at 4 °C). After passed through a 0.2 µm filter, supernatants were lyophilized and store at 4 °C until use.

*Antitumor Studies*: B16‐OVA or B16F10 tumor cells were subcutaneously implanted into the flank of mice on day 0. Then, mice were treated as described in the relevant figure legend. Tumors were measured by digital caliper, and volumes (*V*) were calculated as *V* = π/6 × length × width^2^. Mice were euthanized when tumor length exceeded 2 cm or when the animals showed signs of pain or distress such as immobility, a hunched posture or a lack of eating.

*Statistical Analyses*: Experiments were assessed for significance using one‐way ANOVA, followed by the Bonferroni post‐test, unless noted otherwise. All statistical analyses were done with GraphPad Prism 6.0 (GraphPad Software). A significance threshold of *P* < 0.05 was applied to all comparisons.

## Conflict of Interest

The authors declare no conflict of interest.

## Supporting information

Supplementary
