# Supplementary material for: Turning the Old Adjuvant from Gel to Nanoparticles to Amplify CD8+ T Cell Responses
Source: Adv Sci (Weinh). 2017 Nov 9;5(1):1700426. doi: 10.1002/advs.201700426 (PMC5770685; doi:10.1002/advs.201700426)
Supplement: Supplementary file 1 — Supplementary [file ADVS-5-na-s001.pdf]

Copyright WILEY-VCH Verlag GmbH & Co. KGaA, 69469 Weinheim, Germany, 2017.

# Supporting Information

for *Adv. Sci.*, DOI: 10.1002/advs.201700426

# Turning the Old Adjuvant from Gel to Nanoparticles to Amplify CD8$^+$ T Cell Responses

*Hao Jiang, Qin Wang, Lin Li, Qin Zeng, Hanmei Li, Tao Gong, Zhirong Zhang, and Xun Sun**

Copyright WILEY-VCH Verlag GmbH & Co. KGaA, 69469 Weinheim, Germany, 2017.

# Supporting Information

## Turning the old adjuvant from gel to nanoparticles to amplify CD8$^+$ T Cell Responses

*Hao Jiang, Qin Wang, Lin Li, Qin Zeng, Hanmei Li, Tao Gong, Zhirong Zhang and Xun Sun**

A

| | Size(nm) | PDI | Zeta(mV) | pH |
|---|---|---|---|---|
| AlO(OH) | 1144±108.77 | 0.742±0.178 | 14.63±0.31 | 7.11±0.11 |
| Alhydrogel2% | 736±10.27 | 0.245±0.010 | 16.9±0.77 | 6.20±0.04 |

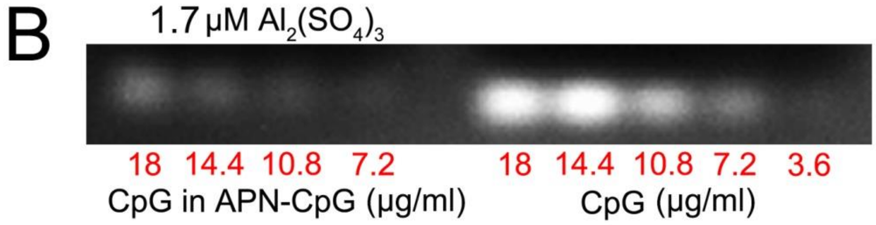


**Figure S1.** (A) Size, PDI, zeta potential and pH of AlO(OH) and Alhydrogel2%. AlO(OH) refers to the same compositions of APNs but without PpAS. (B) Encapsulation of different amounts of CpG in APNs containing 1.7 μM Al2(SO4)3 was measured using agarose gel electrophoresis.

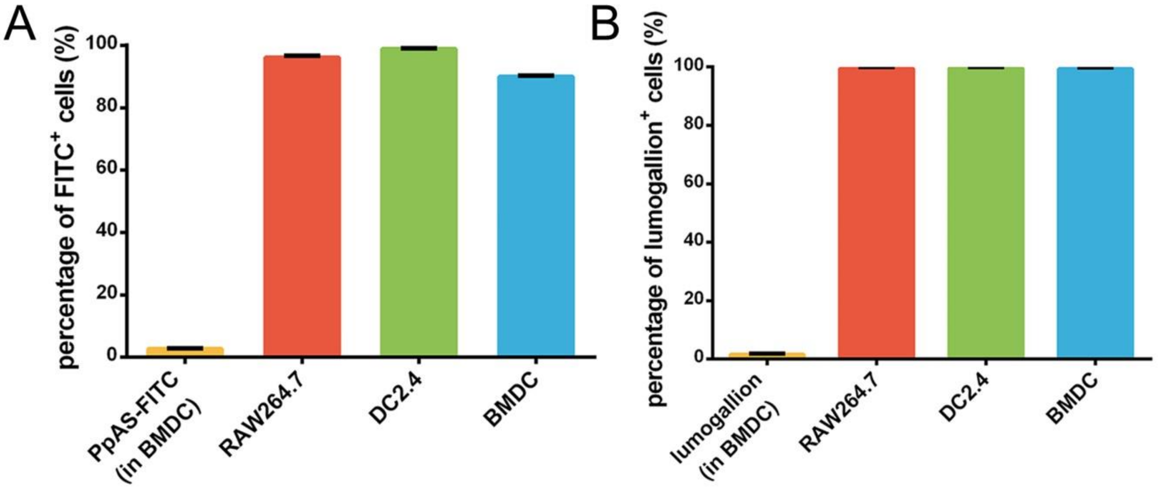


**Figure S2.** Internalization of APNs containing FITC-PpAS (A) or APNs stained with lumogallion (B) by two types of DCs (DC2.4 and BMDCs) and by macrophages.

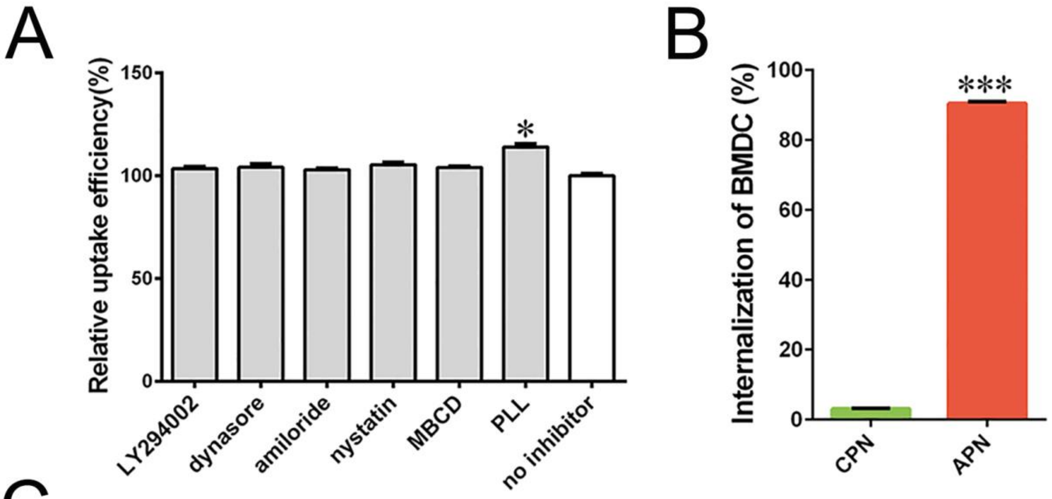


C

| | Size(nm) | PDI | Zeta(mV) | pH |
|---|---|---|---|---|
| CPN | 83.58±2.27 | 0.091±0.009 | -11.11±0.74 | 7.58±0.12 |
| APN | 85.36±1.46 | 0.191±0.005 | -9.35±0.23 | 7.29±0.04 |

**Figure S3.** (A) Poly-L-lysine (PLL) increased APN uptake by DC2.4 cells, perhaps because of electrostatic interactions between the negatively charged APNs and positively charged PLL. Other inhibitors showed no effect on APN uptake. (B) Replacing the AlO(OH) core of APNs with calcium phosphate (CPNs) led to much lower internalization of FITC-APN by BMDCs. (C) Size, PDI, zeta potential and pH of CPN, which are similar to APN. Results for each formulation were compared to those for the 'no inhibitor' control. *$P < 0.05$; MBCD, methyl-β-cyclodextrin.

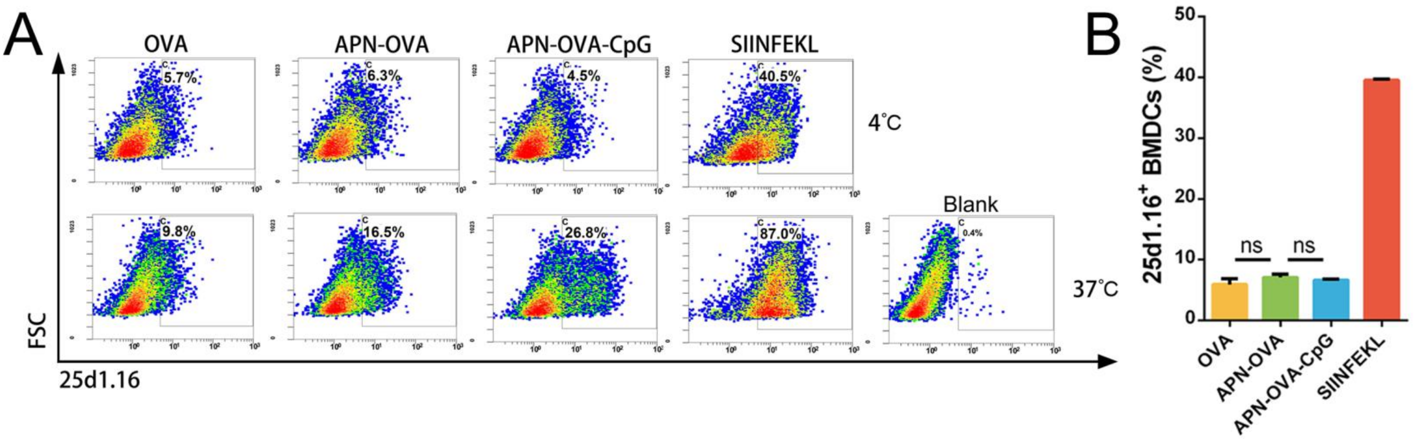

**Figure S4.** (A) BMDCs were cultured for 2 h at 4 or 37 °C with 1.5 μg free or APN-encapsulated OVA, then supernatants were replaced with fresh complete RPMI 1640 medium and cultures were incubated a further 12 h. H2-Kb-SIINFEKL complexes on the surface of BMDCs were detected using PE-labeled monoclonal antibody 25d1.16. Free SIINFEKL peptide can bind extracellularly to H2-Kb, but OVA cannot. Instead, OVA must be processed intracellularly, which is inhibited by incubation at 4 ˚C. (B) The percentage of BMDCs positive for binding to the monoclonal antibody 25d1.16 was quite low when cells were cultured with the indicated formulations at 4 °C. ns, not significant.

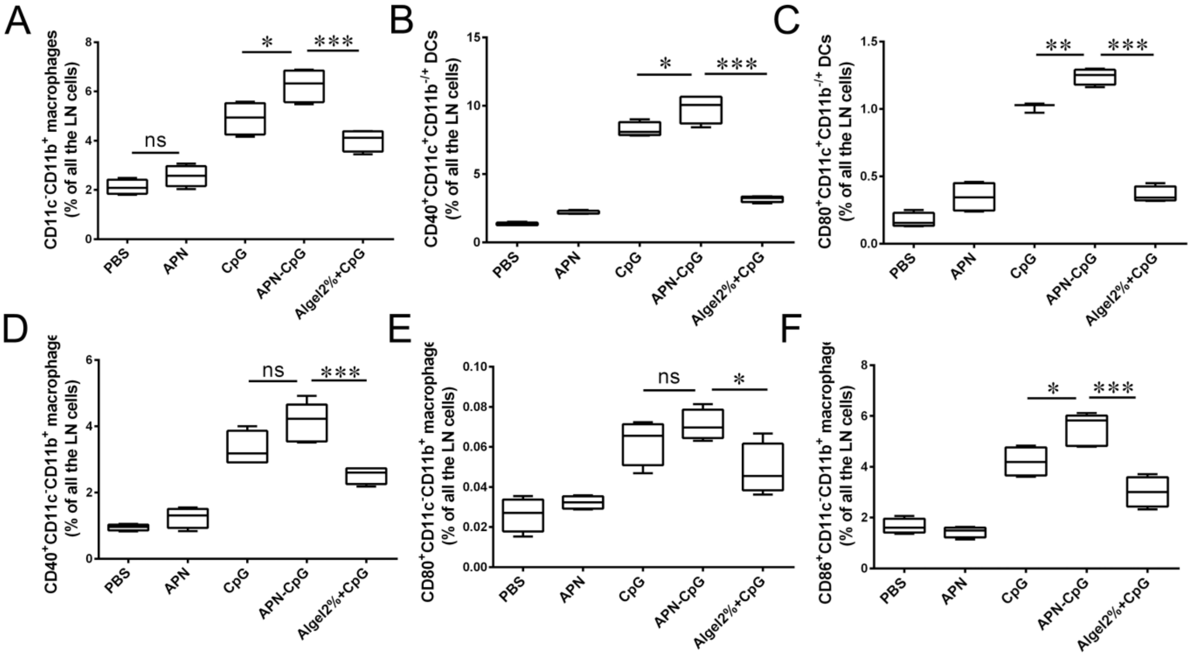


**Figure S5.** (A) $CD11c^{-}CD11b^{+}$ macrophages in total lymph node cells were determined using flow cytometry. Quantification of the percentage of mature $CD11c^{+}CD11b^{-/+}$ DCs in total lymph node cells, based on surface expression of CD40 (B) and CD80 (C). APN-CpG increased the expression of CD40 and CD80 to a significantly greater extent than Algel2% + CpG or OVA + CpG did. (D-F) APN-CpG increased the expression of co-stimulatory molecules (CD40, CD80, and CD86) on $CD11c^{-}CD11b^{+}$ macrophages to a significantly greater extent than Algel2% + CpG did, and to a similar extent as OVA + CpG did.

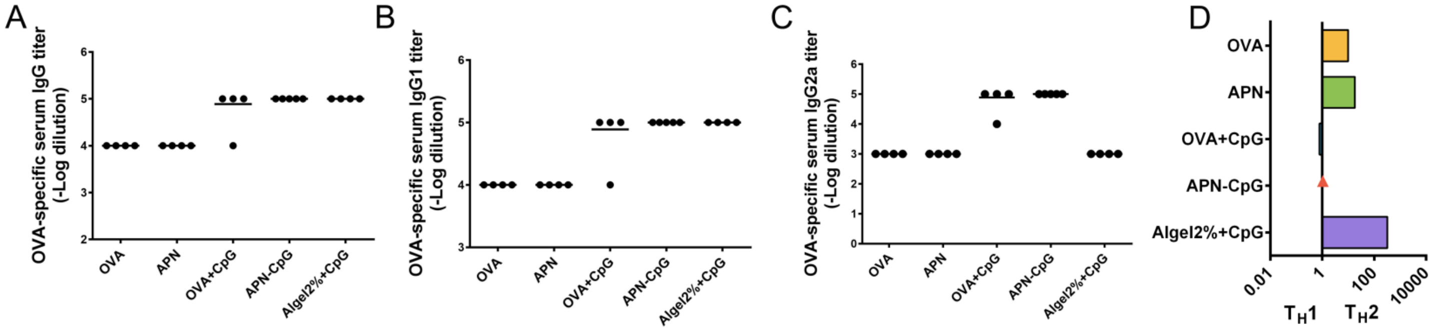


**Figure S6**. C57BL/6 mice were primed on the footpad on day 0 with APNs (with or without 0.45 μg CpG), 1.5 μg OVA (with or without 0.45 μg CpG), or Algel2% with 0.45 μg CpG. Then boost injections were administered on day 7. Serum of vaccinated mice was collected on day 14 and analyzed for total anti-OVA IgG (A) and the subtypes IgG1 (B) and IgG2a (C), as well as for the ratio of Ig1/IgG2a isotypes (D).

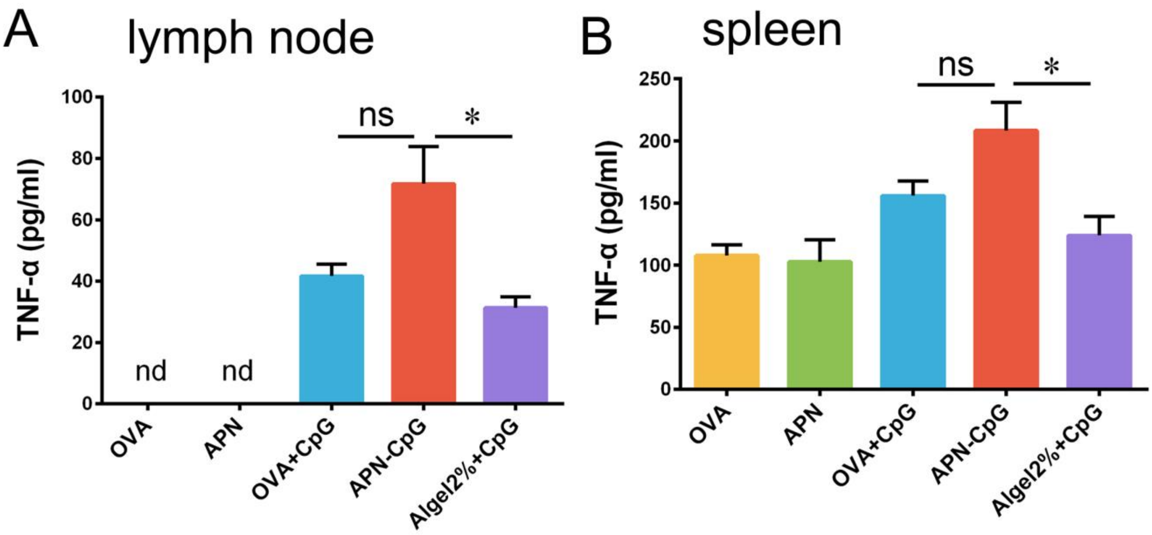


**Figure S7.** C57BL/6 mice were vaccinated in the footpad on day 0 with APNs (with or without 0.45 μg CpG), 1.5 μg OVA (with or without 0.45 μg CpG), or Algel2% with 0.45 μg CpG. A boost injection was performed on day 7 with the same doses of OVA and CpG. Mice were sacrificed on day 14, and popliteal lymph nodes and spleens were harvested and homogenized into cell suspensions. Lymph cells (A) and splenocytes (B) were cultured for 60 h in the presence of 2 μg/ml SIINFEKL, and production of TNF-α was determined using ELISA.
